# Supplementary figures and images for: Parameter calibration of the discrete element simulation model for soaking paddy loam soil based on the slump test
Source: PLoS One. 2023 Jun 2;18(6):e0285428. doi: 10.1371/journal.pone.0285428 (PMC10237372; doi:10.1371/journal.pone.0285428)

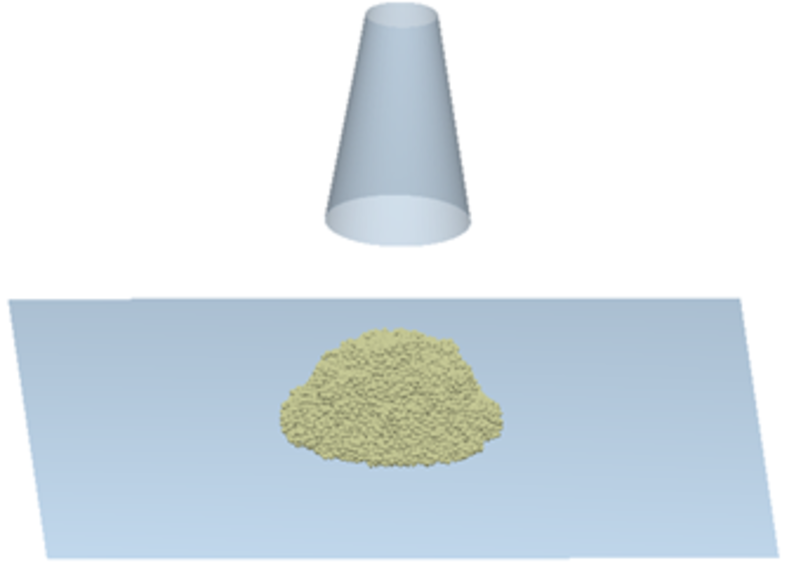

Supplement: S1 Fig — (TIF) [file pone.0285428.s001.tif]
