# Supplementary material for: Parameter calibration of the discrete element simulation model for soaking paddy loam soil based on the slump test
Source: PLoS One. 2023 Jun 2;18(6):e0285428. doi: 10.1371/journal.pone.0285428 (PMC10237372; doi:10.1371/journal.pone.0285428)
Supplement: S1 File — (DOCX) [file pone.0285428.s002.docx]

**Supporting information**

1. Soil moisture content measurement in paddy fields

The samples were put into aluminium boxes, covered and weighed on a balance; the lids were removed and the samples and boxes were put into an oven and baked at a temperature of 105°C to a constant amount; the dried samples and boxes were taken out, covered and cooled to room temperature in a desiccator and the dry soil mass was weighed; the moisture content of the soil was measured and calculated for five samples and the average moisture content was measured to be 32%.

The formula to calculate moisture content (also known as water content) is:

$$\omega=\frac{G_{1}-G_{2}}{G_{2}}$$

Where:$\omega$ is Moisture content，%. *G_1_* is the weight of the sample before drying. *G_2_* is the weight of the sample after drying.

| No. | wet weight / g | dry weight/g | Moisture content /% |
| --- | --- | --- | --- |
| 1 | 46.70 | 35.52 | 31.48 |
| 2 | 36.54 | 27.59 | 32.44 |
| 3 | 43.05 | 32.55 | 32.26 |
| 4 | 41.83 | 31.69 | 31.99 |
| 5 | 32.47 | 24.61 | 31.94 |
| average |  |  | 32.02 |

1. Aual slump measurement

The actual slump of the paddy soil measured according to the slump measurement method is shown in the table below

| No. | slump value / mm |
| --- | --- |
| 1 | 178 |
| 2 | 175 |
| 3 | 180 |
| 4 | 176 |
| 5 | 178 |
| average | 177.4 |

1. Simulation of slump test

The slump simulation process is shown in the following diagram


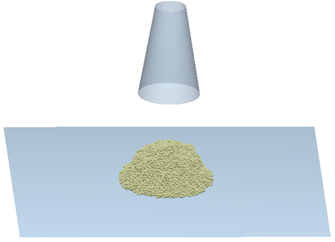


4. Slump simulation measurement

To verify the optimisation results, slump simulation tests were performed using the optimised parameter combinations and the results are shown in the table below.

| No. | surface energy/（J/m^2^） | Soil shear  modulus/Mpa | soil-iron plate static fri-ction coefficient | Slump value/  mm | Slump error/% |
| --- | --- | --- | --- | --- | --- |
| 1 | 3.257 | 0.709 | 0.701 | 182.92 | 3.11 |
| 2 |  |  |  | 177.20 | 0.11 |
| 3 |  |  |  | 175.20 | 1.24 |
| 4 |  |  |  | 173.66 | 2.11 |
| 5 |  |  |  | 183.77 | 3.63 |
| average |  |  |  | 178.55 | 2.04 |
