# Supplementary material for: Parameter calibration of the discrete element simulation model for soaking paddy loam soil based on the slump test
Source: PLoS One. 2023 Jun 2;18(6):e0285428. doi: 10.1371/journal.pone.0285428 (PMC10237372; doi:10.1371/journal.pone.0285428)
Supplement: Attachment — Submitted filename: Response to viewers.docx [file pone.0285428.s003.docx]

Dear Editor and reviewers

Thank you for giving us an opportunity to revise our manuscript. We appreciate the time and effort you put into this manuscript, and your suggestions have helped us improve our work. According to the instructions in your letter, we have uploaded the revised manuscript file. At the same time, we uploaded an annotated manuscript file highlighting the changes made to the manuscript.

We have studied the reviewers’ comments carefully. According to the reviewers’ detailed suggestions, we have carefully revised the original manuscript. All revised portions are marked in red in the manuscript, which we would like to submit for your kind consideration.

We hope that the revised manuscript selects for publication in the PloS ONE

Sincerely

Academic Editor：

Review Comments to the Author

*1.Please ensure that your manuscript meets PLOS ONE's style requirements, including those for file naming.* *The PLOS ONE style templates can be found at https://journals.plos.org/plosone/s/file?id=wjVg/PLOSOne_formatting_sample_main body.pdf and https://journals.plos.org/plosone/s/file?id=ba62/PLOSOne formatting sample title authors affiliations.pdf*

Response: We have confirmed that this manuscript complies with the publication requirements of PLOS ONE.

*2. In your Methods section, please provide additional information regarding the permits you obtained for the work. Please ensure you have included the full name of the authority that approved the field site access and, if no permits were required, a brief statement explaining why*

Response: No permits were required for this research project as it did not involve access to protected or sensitive areas, species, or human subjects and did not violate applicable laws or regulations in the country or region where the research was conducted.

3. *PLOS requires an ORCID ID for the corresponding author in Editorial Manager on papers submitted after December 6th, 2016. Please ensure that you have an ORCID iD and that it is validated in Editorial Manager. To do this, go to ‘Update my Information’ (in the upper left-hand corner of the main menu), and click on the Fetch/Validate link next to the ORCID field. This will take you to the ORCID site and allow you to create a new iD or authenticate a pre-existing iD in Editorial Manager. Please see the following video for instructions on linking an ORCID iD to your Editorial Manager account:* [*https://www.youtube.com/watch?v=_xcclfuvtxQ*](https://www.youtube.com/watch?v=_xcclfuvtxQ)

Response: I have verified my ORCID ID in the Editorial Manager.

I would like to add a statement regarding financial disclosure. This study was supported by the National Natural Science Foundation of China (Grant No. 52005162). The funding organization had no involvement in the study design, data collection and analysis, decision to publish, or preparation of the manuscript.

*Reviewer #1:*

*Comments to the Author*

*The manuscript needs further great improvement. There is currently no discussion section in the manuscript, and it is suggested that additional discussion be added to further explain the experimental results*.

Response: Thank you for your comments. I have added the discussion section to Chapter 4, 'Discussion and Conclusion', as suggested. I appreciate your input and will continue to work on improving the manuscript

*1. Introduction: The introduction section needs further improvement. For example, the author lists the results of six references, but does not make a comprehensive summary associated with the results. It's not clear. Authors need to give a discussion and evaluation associated with the research progress on development of high-performance paddy field pulping machine and the interaction mechanism between the implements and the soil. That is, what problems have been solved by existing research and what problems have not been solved? Which specific problems should be solved in this paper?*

*In the manuscript, readers may not be clear about the difference between this study and other researches, that is, where is the innovation?*

Response: Thank you for your comments. We will be happy to make changes and additions to the text in response to helpful comments from the reviewers. I have separated my responses to the reviewers` comments according to several categories in order to achieve an integrated approach in my responses.

1. As per your suggestion, I have included a summary and discussion of this part of the research in the literature review section of the manuscript.

2. As per your suggestion, I have added a discussion on the development of a high-performance paddy field pulping machine and the latest research on the mechanisms of interaction between the implements and the soil in the introduction. In this section, we provide an analysis of the problem to be addressed and outline the work carried out in this paper to tackle this issue.

3. As per your recommendation, we have incorporated innovative points in the discussion and conclusion section. Specifically, we calibrated the parameters of a discrete element simulation model for the clay loam soil of paddy fields prior to pulping to address the current lack of an accurate soil model and to advance the development and application of the discrete element method in paddy pulping research. The study aims to provide a precise base model for investigating the interaction mechanism between pulping parts and paddy field soil during the paddy pulping process, thereby enabling significant progress in the field.

*2. In section for “2.1 determination of basic physical parameters”, if only one kind of soil is taken, this study is of little significance. It is necessary to add investigation to consider the effects of soil type or soil structure.*

Response: Thank you for your comments. It is worth noting that there are numerous types of soils used for rice cultivation and their physical properties can vary significantly. Thus, studying different types of paddy soils is necessary. However, the clay loam soil investigated in this research is a representative soil for rice growth in the rice-wheat rotation area. The calibration of its discrete element parameters is of significant reference value for simulating the rice pulping process by rice pulpers in this region.

*3. The authors should add some introduction to the methods in the manuscript, such as Plackett-Burman test, the steepest climb test, the Box-Behnken test.*

Response: Thank you for your comments, Following your suggestion, I have added an introduction to each part of the test.

*4. Some of the experimental schemes in the manuscript should be explained in more detail:
(1) How to determine the value range of 9 parameters to be calibrated according to the literature?*

Response: This part has been introduced in the paper to supplement the soil properties of the references found to be similar to the soil properties studied in this study, and the range of values for the soil parameters calibrated in the course of the previous study can be derived for this paddy field soil by synthesizing and organizing them.

*(2) How is the combination of parameter levels involved in the experimental protocol determined in table 2.*

Response: Thank you for your comments. The method for determining the combination of parameter levels has been supplemented in the paper. We used Design Expert software to conduct a Plackett-Burman design and obtain the parameter combinations listed in Table 2.

*(3) Are the values of the 4 levels and their corresponding parameters fixed in table 4?*

Response: Yes, the 4 levels` corresponding parameters are fixed in Table 4. Table 4 shows the design scheme and results of the steepest ascent test. the rest of the parameters have no significant influence on the test index to take the middle value of their respective value range.

*(4) According to Table 5, there are 27 schemes can be formed, why only 17 schemes are selected in the study, and the values of parameters b, c and h are the same for schemes after No.14*

Response：The Box-Behnken test was coded for three factors and three levels, and the number of scenarios generated when only one Block and five centroids were available was 17 when the Box-Behnken test scenarios were generated using Design-Expert software.

The values of parameters b, c, and h are the same for the scenarios after No. 14. Because the error of the test scenario needs to be estimated, so the test is repeated five times for the center point to improve the accuracy and reliability of the test results.

*5. The slump error of paddy soil is used as a test index to calibrate the discrete element simulation parameters in the manuscript.*

*The guiding significance for the study of interaction mechanism between the implements and the soil, or for t development of high-performance paddy field pulping machine is not clear.*

Response：Thank you for your suggestion, As per your suggestion, I have added an explanation of this section in the paper's introduction. This test is a basic model study to provide an effective tool and means to study the interaction mechanism between pulping components and paddy soil during paddy beating, so as to optimize the parameters of paddy beating device to improve its working quality and efficiency. In this paper, a discrete element model of a typical paddy soil is calibrated to facilitate the application and development of the discrete element method in the field of paddy pulping research.

*Reviewer #2:*

*Review Comments to the Author*

*1.There were several typos, grammatical errors, and incomplete sentences. Authors are advised to edit the manuscript for better clarity.*

Response：Thank you for your language editing suggestions. We have had the article touched up by a professional, which has helped improve the quality of our work. Your feedback is very valuable and we appreciate your help in improving the manuscript.

*2.Add better and high quality images.*

Response：Thanks for your suggestion. I have replaced the original images with higher quality ones that are clearer.

3.Recent literature is sufficiently cited. Please check all the references one by one to avoid any mistakes in-text citations.

Response：Thank you for your feedback. I have carefully checked and verified all the references cited in the text. I appreciate your suggestion to ensure the accuracy of in-text citations.

I tried my best to improve the manuscript and made some changes marked in red in revised paper. We appreciate for editors and reviewers’ warm work earnestly, and hope the correction will meet with approval. Once again, thank you very much for your comments and suggestions.
